# Geometric pacing: external reference support as a principle of physiological stabilization in aging and pre-pathological states

**DOI:** 10.3389/fnetp.2026.1801602

**Published:** 2026-05-20

**Authors:** Alejandro Carballo

**Affiliations:** 1 Nässjö Läkarhus, Region Jönköping County, Nässjö, Sweden; 2 Futurum - Academy for Health and Care, Region Jönköping County, Jönköping, Sweden

**Keywords:** aging, autonomic regulation, coordination dynamics, cross-system coupling, external cueing, network physiology, persistent physical symptoms, physiological stabilization

## Abstract

**Background:**

Cardiac pacemakers exemplify how minimal external reference signals can restore functional stability in dysregulated biological systems without correcting the underlying etiology. This epistemological model suggests broader applications for understanding physiological support mechanisms beyond the heart.

**Objective:**

This Perspective proposes geometric pacing as a general physiological principle whereby externally provided rhythmic, spatial, or proprioceptive references support self-organization in systems characterized by regulatory instability rather than structural pathology.

**Framework:**

Drawing on coordination dynamics, autonomic regulation, polyvagal perspectives, and evidence from cueing interventions in neurological rehabilitation, the article develops a conceptual framework applicable to aging, persistent physical symptoms, and pre-pathological states marked by fatigue, autonomic lability, reduced adaptive reserve, and inefficient multisystem coordination. The argument is explicitly situated within Network Physiology by treating stabilization as an emergent property of interactions across autonomic, sensorimotor, interoceptive, and postural subsystems operating across multiple temporal scales.

**Conclusion:**

Geometric pacing is proposed as a rate-modifying, non-specific support mechanism that may reduce regulatory uncertainty and energetic cost by constraining unstable system dynamics toward more coherent and efficient states. Rather than targeting isolated organs, the framework addresses organism-level coordination and cross-system integration. The article deliberately avoids proposing specific devices or protocols, instead articulating a principle intended to invite interdisciplinary investigation within the broader field of Network Physiology while preserving flexibility for future clinical, environmental, rehabilitative, or technological implementations.

## 1 Introduction: pacemakers as an epistemological template

The adoption of cardiac pacemakers represents one of medicine’s most elegant solutions to a complex problem. Their clinical success was established not because we fully understood the molecular basis of conduction disorders, but because a minimal and reliable temporal reference could restore functional stability in a system whose intrinsic rhythm generation had become unreliable. Importantly, pacing does not replace intrinsic physiology; it supports self-organization by preventing the collapse of coordination.

This historical precedent raises a broader question: are there other physiological systems in which dysfunction reflects loss of internal reference rather than loss of capacity, and could such systems benefit from analogous external reference support? The present Perspective argues that the answer may be affirmative and develops a conceptual framework we term geometric pacing. Crucially, the relevant inputs need not be periodic: as will be discussed, non-periodic stochastic signals—random sub-threshold noise—can also stabilize physiological function through the mechanism of stochastic resonance, extending the framework beyond the rhythmic domain from its outset.

The central claim is that some age-related and pre-pathological states may be understood not primarily as lesions of isolated organs, but as disturbances in the coordination among physiological subsystems. From this viewpoint, external reference support may help stabilize global organismic function without substituting for intrinsic control.

This framing is especially relevant to Network Physiology, a field concerned with how physiological systems and subsystems dynamically interact, synchronize, and integrate across levels and time scales to generate functional states in health and disease (Bashan et al., 2012; Ivanov, 2021). In this context, geometric pacing is not a local intervention model but a hypothesis about how external constraints may influence network-level physiological organization.

## 2 Theoretical foundations

### 2.1 Coordination dynamics and self-organization

Bernstein’s foundational work on movement coordination established that the central nervous system does not control individual degrees of freedom independently but organizes them into functional synergies—task-specific, temporarily assembled units that reduce control complexity (Bernstein, 1967). Kelso and colleagues subsequently demonstrated that these synergies exhibit self-organizing dynamics: they emerge spontaneously from cooperative coupling among components and can switch abruptly between stable patterns when control parameters change (Kelso, 1984; Haken et al., 1985; Kelso, 1995).

The Haken-Kelso-Bunz model formalized these observations mathematically, demonstrating that coordination patterns can be described as attractors in a low-dimensional dynamical system. Critically, this model showed that external perturbations or rhythmic inputs can stabilize, destabilize, or shift between coordination patterns—a principle with direct relevance to the present proposal.

### 2.2 Autonomic regulation and multisystem flexibility

Autonomic regulation is a central component of the framework developed here because it links cardiovascular, respiratory, affective, interoceptive, and behavioral processes into a distributed regulatory architecture rather than a single isolated control loop (Bashan et al., 2012; Ivanov, 2021). Disturbance at this level may therefore propagate across systems and contribute to diffuse but functionally meaningful instability, which is precisely the class of phenomena the present framework addresses.

The empirical observations that matter for this argument are well established and do not depend on any single interpretive framework. Heart rate variability, particularly respiratory sinus arrhythmia, declines with age and is associated with reduced adaptive capacity (Byrne et al., 1996). Frail older adults are significantly more likely to exhibit autonomic dysfunction (Debain et al., 2023). Autonomic symptoms are common in older adults and are associated with reduced health-related quality of life (Renno-Busch et al., 2021). Taken together, these findings support the more limited claim on which this manuscript depends: that autonomic flexibility, indexed by HRV and related measures, indexes a dimension of regulatory capacity that declines with age and is a plausible substrate for network-level stabilization.

Polyvagal theory (Porges, 1995; Porges, 2007) has offered one interpretive overlay on this empirical core, proposing a hierarchical organization of autonomic states with specific anatomical and evolutionary commitments. That theory is currently the subject of substantive debate. A coordinated critique by thirty-nine autonomic physiologists and comparative biologists has argued that specific neuroanatomical, measurement, and evolutionary claims of polyvagal theory are not defensible on current evidence (Grossman et al., 2026), and Porges (2026) has published a detailed rebuttal in the same issue. The present manuscript does not rely on resolving this debate. Its argument requires only the empirical claims stated above, which hold independently of the contested anatomical and evolutionary specifics. Where the polyvagal vocabulary is clinically familiar to readers, it may be useful as a descriptive overlay; where the underlying mechanisms are contested, the framework developed here is deliberately built on the primary empirical findings rather than on those mechanisms.

### 2.3 Aging, allostasis, and regulatory inefficiency

The hallmarks of aging framework identifies interconnected processes that characterize biological aging, including altered intercellular communication and chronic inflammation (López-Otín et al., 2013; López-Otín et al., 2023). While molecular in focus, this framework recognizes that aging involves progressive loss of physiological integrity—a formulation consistent with the coordination dynamics perspective that regulatory coordination, not merely component function, deteriorates with age.

The allostatic load model complements these perspectives by describing the cumulative cost of chronic stress on physiological systems (McEwen and Stellar, 1993; Sterling and Eyer, 1988). Prolonged allostatic activation can produce persistent regulatory deviation—a state in which the organism maintains suboptimal organization at elevated energetic cost, even after the original stressor has resolved. In this sense, regulatory inefficiency may be regarded as a network-level phenotype: the organism still functions, but with lower flexibility, higher effort, and greater vulnerability to perturbation. The theoretical traditions informing this framework are summarized in Table 1.

**TABLE 1:** Theoretical foundations supporting the geometric pacing framework.

Theoretical tradition	Key concepts	Relevance to geometric pacing
Coordination Dynamics (Kelso, 1995)	Self-organization, attractors, phase transitions, metastability	External inputs can stabilize coordination patterns
Autonomic flexibility framework (HRV tradition; polyvagal among interpretive overlays, currently contested — Grossman et al., 2026; Porges, 2026)	HRV, respiratory sinus arrhythmia, age-related decline in flexibility, autonomic dysfunction in frailty	Autonomic flexibility as a substrate for pacing; argument relies on the empirical core rather than on contested specifics
Allostasis (Sterling and Eyer, 1988)	Allostatic load, regulatory deviation, energetic cost	Inefficient states may be modified by external reference
Hallmarks of Aging (López-Otín et al., 2023)	Altered intercellular communication, inflammaging, loss of homeostasis	Aging involves coordination failure, not just component failure
Network Physiology (Bashan et al., 2012; Ivanov, 2021)	Physiological networks, cross-system coupling, multiscale dynamics	Stabilization can emerge from improved interactions across subsystems

Each theoretical tradition contributes distinct concepts to understanding how external references might support physiological self-organization.

## 3 Geometric pacing as a network physiology framework

We define geometric pacing as the provision of external references—rhythmic, spatial, proprioceptive, or stochastic—that scaffold self-organization in systems with impaired internal coordination. The term ‘geometric’ emphasizes that relevant references may be spatial as well as temporal, and that the class of effective inputs includes both periodic signals and non-periodic noise operating at sub-threshold amplitudes, extending the principle well beyond the purely rhythmic domain of cardiac pacing.

Analogous to cardiac pacing, geometric pacing has four defining properties:

1. External: the reference originates outside the system’s intrinsic control mechanisms.
2. Indirect: the intervention is supportive rather than substitutive; the organism remains self-regulating.
3. Systemic: the principal effect is on coordination patterns rather than on isolated components.
4. Rate-modifying: the impact is expected to accumulate over time by reducing drift, variability, and regulatory inefficiency.

No central controller is assumed. The organism maintains its self-organizing capacity; geometric pacing operates by constraining the space of possible states toward more stable and efficient configurations.

What makes this explicitly a Network Physiology concept is that the target is not an individual symptom, organ, or pathway, but the dynamic coupling among subsystems (Bashan et al., 2012; Ivanov, 2021). The relevant substrate may include interactions among posture, gait, respiration, autonomic tone, vestibular signals, sensory orientation, and interoceptive processing. In such cases, physiological stabilization is best understood as an emergent consequence of improved network integration.

Loss of internal reference is therefore framed here not simply as local failure, but as partial disintegration of organism-level coordination.

### 3.1 Differentiation from adjacent frameworks

Because the intuitions motivating geometric pacing overlap with several established frameworks, it is worth stating explicitly what the concept adds and what it does not. Three adjacent frameworks deserve direct comparison: sensory substitution, environmental enrichment, and the loss of complexity hypothesis.

Sensory substitution, as introduced by Bach-y-Rita and colleagues, uses an intact modality to convey world-specifying information that a damaged modality can no longer deliver (Bach-y-Rita et al., 1969; Bach-y-Rita and Kercel, 2003). The defining feature is that the substituting input carries the informational content of the missing channel—the tactile array on the back or tongue encodes a visual scene. Geometric pacing requires the opposite: the external reference must be informationally thin. A metronome beat, a subthreshold noise floor, or a geometrically simple postural cue works precisely because it does not specify content but imposes temporal or spatial structure. A direct empirical consequence follows: if geometric pacing and sensory substitution were the same mechanism, stripping the reference of semantic content should abolish the effect. The framework predicts the opposite—effects should be preserved, or enhanced, when the reference is informationally minimal but structurally reliable.

Environmental enrichment, in the Rosenzweig-derived tradition, acts through increased variety, novelty, and multimodal richness of stimulation, and operates principally through neuroplastic remodelling—synaptogenesis, dendritic branching, and, in some paradigms, adult hippocampal neurogenesis (Balietti and Conti, 2022). Its characteristic dose–response profile is cumulative and structural, with effects emerging over weeks. Geometric pacing inverts both features: it predicts that minimal, repetitive, structurally constrained inputs are sufficient, and that stabilization should appear on coordination variables within seconds to minutes of exposure, with longer-term recalibration superimposed on this fast effect. Empirically, the two frameworks can therefore be distinguished by the information-richness of the input and by the latency of the response.

The loss of complexity hypothesis (Lipsitz and Goldberger, 1992; Lipsitz, 2004; Manor and Lipsitz, 2013) operates at a different epistemic level. It is a descriptive characterization of age-related dysregulation at the level of single-system output: healthy physiological dynamics exhibit complex, multiscale, fractal-like variability, and aging erodes that complexity. Geometric pacing is not a competitor to this hypothesis but a prescriptive complement. Loss of complexity identifies what age-related dysregulation looks like in the output of individual systems; geometric pacing proposes a class of intervention acting on coupling between systems. A consequence of this division of labor is that geometric pacing does not strongly predict the restoration of within-system complexity. It predicts the stabilization of inter-system coupling, which may or may not be accompanied by recovery of fractal structure in any given single-system signal.

**FIGURE 1:**
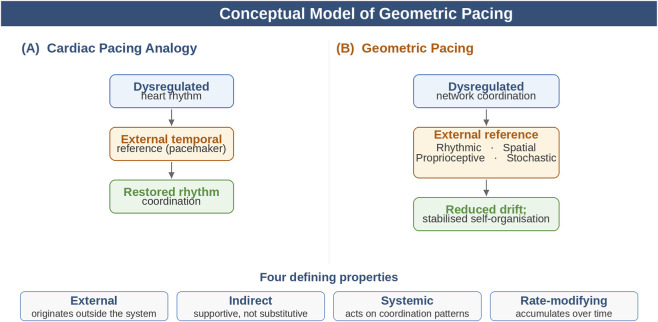
Conceptual model of geometric pacing. **(A)** Cardiac pacing analogy; **(B)** extension to geometric pacing with rhythmic, spatial, and proprioceptive references.

Table 2 summarizes these distinctions. The differentiation also clarifies the role of stochastic resonance (Section 6.5): stochastic resonance is a subclass of geometric pacing because the noise carries no information content and acts on threshold dynamics; it is not a form of sensory substitution because no channel is replaced, and it is not environmental enrichment because it is minimal and structured rather than rich and varied (Figure 1).

**TABLE 2:** Differentiation of geometric pacing from adjacent frameworks.

Axis	Sensory substitution	Environmental enrichment	Loss of complexity	Geometric pacing
Input content	Information-rich; encodes missing channel	Rich, novel, multimodal	Not applicable (descriptive framework)	Informationally minimal; structurally constrained
Level of action	Perceptual channel recruitment	Neuroplastic remodelling (synapses, neurogenesis)	Single-system output variability	Inter-system coupling and coordination
Temporal signature	Seconds–minutes for detection; months for skilled use	Weeks; cumulative and structural	Describes slow age-related decline	Fast on coupling (seconds–minutes); slow on attractor landscape (hours–weeks)
Distinguishing empirical prediction	Effect destroyed by removing informational content	Effect depends on variety; plasticity markers co-vary	Predicts loss of fractal variability, not a recovery mechanism	Effect preserved under informationally minimal inputs; measured at cross-system coupling level
Relation to geometric pacing	Distinct; different class of input	Distinct; different dose and mechanism	Complementary; different epistemic level	—

Stochastic resonance interventions (Section 6.5) fit within the geometric pacing column and are therefore a subclass of this framework rather than a separate entry.

## 4 Empirical parallels: external cueing in neurological rehabilitation

The strongest empirical parallels come from cueing interventions in Parkinson’s disease and related movement disorders. External rhythmic and visual cues can improve stride length, gait velocity, gait-related mobility, and movement initiation in patients whose motor capacity is partly preserved but whose internally generated movement timing is unreliable (Azulay et al., 2006; Ghai et al., 2018; Nieuwboer et al., 2007; Tedeschi et al., 2024; Zhang et al., 2024). Rhythmic cueing may also recruit alternative cerebellar and sensorimotor pathways that help bypass impaired internal timing mechanisms (Nombela et al., 2013). Key findings from this literature are summarized in Table 3.

**TABLE 3:** Evidence for external cueing in motor rehabilitation.

Study/review	Design	Intervention	Key findings
Ghai et al. (2018)	Systematic review + meta-analysis	Rhythmic auditory cueing in Parkinsonian gait	Stride length g = 0.48; velocity g = 0.27
Nieuwboer et al. (2007)	RCT (RESCUE)	Home-based cueing training	Improved gait-related mobility
Zhang et al. (2024)	Systematic review + meta-analysis	Wearable cueing devices	Efficacy for gait and motor function
Tedeschi et al. (2024)	Scoping review	Multimodal cueing in Parkinson’s disease	5/7 studies reported increased step length

Abbreviations: RCT, randomized controlled trial.

These findings are important not only because they support the plausibility of external reference support, but because they illustrate a broader principle: capacity may remain present even when internal coordination becomes unstable. External cues can transiently reorganize function by stabilizing coupling among sensorimotor subsystems. It must be stated clearly, however, that the Parkinson’s disease cueing literature constitutes proof of principle for this logic in a specific neurological context—not direct evidence for analogous effects in aging or persistent physical symptoms. The extension to those populations is a hypothesis that requires dedicated empirical testing; the cueing evidence establishes only that the underlying mechanism is biologically plausible and measurable.

From a Network Physiology perspective, this suggests that rhythmic or spatial inputs may influence not only local motor output but also broader interactions across cerebellar, basal ganglia, cortical, autonomic, and postural systems. The cue is therefore not merely a trigger; it may act as a network constraint that enables more coherent global behavior.

## 5 Extension to aging and pre-pathological states

### 5.1 Aging as regulatory inefficiency

Older adults frequently report diffuse symptoms—fatigue, heaviness, dizziness, effort intolerance, or reduced steadiness—in the absence of clear organ-specific pathology. Within the present framework, such presentations suggest impaired temporal or geometric coordination at the organism level rather than disease of specific organs. It is important to note, however, that the framework does not claim that coordination disturbance underlies all age-related symptoms: structural pathology, metabolic dysfunction, and pharmacological burden remain primary explanations for many presentations. The claim is more limited—that a meaningful subset of diffuse, pre-pathological functional complaints may reflect coordination failure and could therefore be responsive to external reference support.

This view is supported by evidence that autonomic flexibility declines with age (Byrne et al., 1996), that frail older adults are more likely to exhibit autonomic dysfunction (Debain et al., 2023), and that autonomic symptoms are common in older adults and are associated with reduced health-related quality of life (Renno-Busch et al., 2021). The autonomic nervous system’s role in coordinating multi-organ homeostasis makes it a plausible substrate for network-level stabilization.

### 5.2 Persistent physical symptoms

A recent Lancet review conceptualized persistent physical symptoms as distressing somatic complaints whose link to identifiable pathophysiology often weakens as symptoms persist (Löwe et al., 2024). The authors identify maintaining factors that include persistent inflammation and dysregulation of immune, autonomic, metabolic, and microbiome systems—formulations consistent with the regulatory deviation hypothesis.

Systematic review evidence also indicates adrenergic dysfunction in myalgic encephalomyelitis/chronic fatigue syndrome and fibromyalgia, consistent with autonomic dysregulation across overlapping syndromic presentations (Hendrix et al., 2025). Contemporary theories likewise describe functional somatic syndromes as involving dysregulation in brain-body signaling, with symptoms resulting from complex interactions among autonomic, endocrine, immune, and interoceptive systems rather than peripheral tissue damage alone.

In this light, geometric pacing may be especially relevant to conditions in which the organism has not yet crossed into overt pathology, but is already functioning with diminished adaptive reserve. The aim would not be cure in a conventional sense, but stabilization: lowering regulatory uncertainty, improving coordination efficiency, and supporting recovery of functional flexibility.

### 5.3 A clinical scenario

A concrete example clarifies how a clinician or researcher might identify the pattern the framework targets, and distinguishes it from presentations dominated by organ-specific pathology. Consider a 74-year-old outpatient referred for diffuse fatigue, mild unsteadiness, and effort intolerance with a negative cardiac, neurological, and metabolic work-up. Her resting ECG and echocardiogram are unremarkable, pulmonary function is within age-adjusted norms, and her cognitive screen is intact.

A coordination-oriented assessment adds a small number of inexpensive measures: short-term heart rate variability during paced and spontaneous breathing; stride-time variability during simple and dual-task walking; and static and dynamic posturography. In this patient, the pattern that emerges is instructive: resting single-system measures lie within the age-expected range, but three features appear in combination—reduced respiratory sinus arrhythmia that does not increase appropriately during paced breathing, a rise in stride-time coefficient of variation under dual-task conditions that substantially exceeds the rise seen during single-task walking, and an increase in postural sway under visual deprivation. None of these is diagnostic in isolation, and none points to a specific organ disease. Together, they form a signature consistent with reduced stability of cross-system coupling: the respiratory–cardiac, cognitive–motor, and visual–postural links are each less reliable than would be expected for her single-system capacities.

Within the present framework, this pattern identifies a candidate target for external reference support rather than for organ-directed treatment. Candidate references would be chosen by the domain in which coupling appears weakest. For this patient, a periodic respiratory reference (paced breathing guidance) addresses the respiratory–cardiac link; a rhythmic auditory cue during walking addresses cognitive–motor coupling during dual-task conditions, drawing on the evidence base reviewed in Section 4; and a subthreshold stochastic input to plantar mechanoreceptors, as described in Section 6.5, addresses the visual–postural link. The clinical logic is not that these references treat a disease but that each supplies a stable external constraint in a domain where internal coordination is becoming unreliable. The research question that follows is whether, and in what combinations, such references measurably restore coupling stability—the prediction formalized in Section 9.1.

This scenario is intentionally illustrative rather than prescriptive. It does not recommend a treatment protocol; it shows the decision architecture through which the framework is meant to be applied. A patient whose presentation is dominated by a structural lesion, a metabolic disorder, or a drug effect would be identified and treated within the conventional pathways, and nothing in this framework competes with or displaces that logic. Geometric pacing offers a conceptual vocabulary for the residual category of presentations in which organ-level work-up is negative, multiple single-system measures hover near the lower end of normal, and the signature of instability lies in the relationships between systems rather than in any one of them.

## 6 Proposed mechanistic pathways

While deliberately avoiding specific implementation proposals, several candidate mechanisms may link external references to physiological stabilization. These mechanisms operate at distinct but interacting levels: the neuronal level (oscillatory entrainment, attractor reshaping), the systemic level (autonomic cascade effects, cross-system coupling), and the behavioral level (reduction of prediction burden, stochastic facilitation of sensorimotor thresholds). Their conceptual separation is analytically useful even though in practice they are likely to co-occur and interact.

### 6.1 Reduction of regulatory uncertainty

When internal reference signals degrade, the organism must expend more effort to maintain coordinated behavior. External references may reduce ambiguity and prediction burden, thereby lowering energetic cost. This aligns with predictive processing frameworks that emphasize the brain’s role in minimizing surprise and maintaining functional regulation under uncertainty (Friston, 2010).

### 6.2 Entrainment and phase locking

Rhythmic external inputs may entrain endogenous oscillatory processes and facilitate phase alignment across interacting subsystems. Evidence from neuroimaging demonstrates that rhythmic auditory stimuli modulate beta oscillations in auditory cortex, cerebellum, and sensorimotor regions (Fujioka et al., 2012). Such entrainment may stabilize coordination dynamics that have become variable or inefficient and may support communication through coherence across distributed neural systems (Fries, 2015).

### 6.3 Attractor stabilization

From a dynamical systems perspective, external constraints may deepen preferred attractor states, raise barriers between unstable patterns, or reduce transitions into poorly coordinated states. Prolonged exposure could gradually reshape the intrinsic attractor landscape and produce more durable recalibration, a hypothesis that is testable through time-series analyses of relevant coordination variables.

### 6.4 Cross-system cascade effects

Because breathing, posture, locomotion, autonomic tone, and interoceptive signaling are interdependent, an intervention directed at one domain may propagate through the wider physiological network and modify organism-level behavior (Bashan et al., 2012; Ivanov, 2021). This is precisely the type of cross-system interaction that Network Physiology seeks to characterize.

### 6.5 Stochastic resonance as a complementary class of reference support

Beyond periodic and rhythmic references, non-periodic stochastic inputs—random noise applied at sub-threshold levels—have been shown to improve sensorimotor function and balance in older adults through the mechanism of stochastic resonance (Rogan and Taeymans, 2023). In stochastic resonance, the addition of an optimal level of noise to a subthreshold signal paradoxically enhances signal detection and systems responsiveness.

**FIGURE 2:**
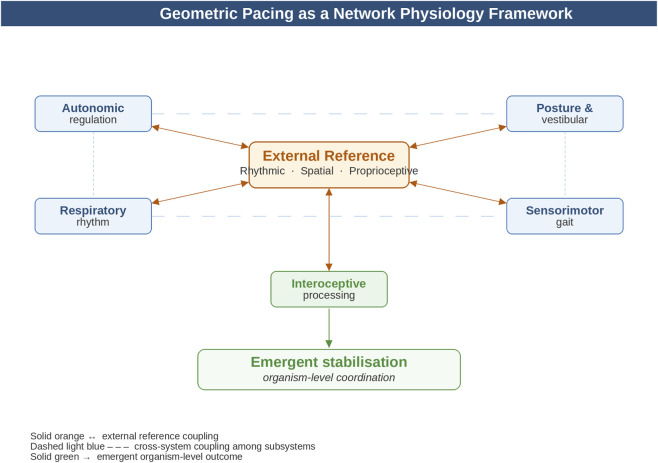
Geometric pacing as a network physiology framework. The external reference interacts bidirectionally with five physiological subsystems; dashed lines indicate cross-system coupling.

This mechanism is conceptually distinct from entrainment and phase-locking but shares the defining logic of geometric pacing: an external input acts not by replacing intrinsic regulatory processes but by modifying the conditions under which self-organization operates. Stochastic resonance interventions may therefore constitute a complementary subclass of geometric pacing—one in which the stabilizing reference is non-periodic rather than rhythmic. This possibility broadens the theoretical scope of the framework and invites systematic comparison of periodic and stochastic external references as regulatory supports in aging populations (Figure 2).

## 7 Why this is not a technical proposal

This Perspective intentionally refrains from specifying devices, parameters, anatomical targets, or treatment protocols—not because implementation is unimportant, but because theoretical coherence should precede specification. The principle may invite contributions from multiple disciplines (neuroscience, engineering, rehabilitation, gerontology, environmental design), clinical claims require evidence whereas conceptual frameworks serve as generative hypotheses, and the possible forms of implementation—behavioral, environmental, wearable, rehabilitative, or biomedical—remain appropriately open.

What remains central is the distinction between principle and protocol. Geometric pacing names a class of mechanism, not a class of device. This distinction is what makes it productive as a research framework.

## 8 Strengths, limitations, and validity of the framework

### 8.1 Strengths

The principal strength of this Perspective is its integrative scope. The geometric pacing framework synthesizes evidence and theory from coordination dynamics, Network Physiology, autonomic flexibility research, allostatic load models, and empirical cueing research into a single generative principle. By framing aging-related regulatory decline as a network-level coordination failure rather than isolated organ pathology, the framework opens a conceptual space that is both scientifically grounded and clinically relevant.

A second strength is the empirical anchor provided by the Parkinson’s disease cueing literature. The existence of well-replicated, quantified effects of external rhythmic and visual cues on gait and motor coordination (Ghai et al., 2018; Zhang et al., 2024) establishes that the underlying principle—external reference support restoring function in systems with preserved but poorly coordinated capacity—is not merely speculative. It is an observed phenomenon in one clinical context whose generalization to aging and pre-pathological states now constitutes a testable hypothesis.

A third strength is the framework’s deliberate non-specificity at the implementation level. Rather than constraining the principle to a particular technology or protocol, the framework preserves flexibility for diverse future instantiations and disciplines. This is appropriate for a foundational Perspective and is consistent with the epistemological role of theory generation in scientific progress.

### 8.2 Limitations

The principal limitation is that geometric pacing remains, at this stage, a conceptual proposal without direct experimental validation in the target populations—older adults and individuals with persistent physical symptoms. The empirical parallels drawn from neurological rehabilitation are suggestive but not sufficient to confirm that analogous mechanisms operate across the range of pre-pathological states described. This limitation is intrinsic to the Perspective format and is explicitly acknowledged in the manuscript’s framing.

A second limitation concerns the operationalization of key constructs. ‘Loss of internal reference,’ ‘regulatory inefficiency,’ and ‘organism-level coordination’ are framed at a level of abstraction that, while theoretically coherent, requires measurement operationalization before experimental testing becomes possible. The research agenda (Section 9) identifies this as a priority, but the gap between the conceptual vocabulary and available instruments remains substantial.

A third limitation is the potential for interpretive overreach. The breadth of the framework—spanning aging, persistent physical symptoms, and pre-pathological states—risks conflating heterogeneous phenomena under a single explanatory principle. The mechanisms proposed (reduction of regulatory uncertainty, entrainment, attractor stabilization, cascade effects, stochastic resonance) are candidate pathways, not established facts, and their relative contributions may differ substantially across conditions and individuals.

### 8.3 Validity of the framework and data interpretation

The conceptual validity of geometric pacing rests on three supporting pillars: (1) the mechanistic plausibility derived from dynamical systems theory and Network Physiology; (2) the cross-disciplinary consistency of the core prediction—that external reference inputs can reorganize coordination in systems with preserved but poorly coupled components; and (3) the empirical evidence from cueing studies in Parkinson’s disease, which provides a proof-of-concept albeit in a specific neurological context.

The interpretation of published findings is, throughout, conservative. Effect sizes from meta-analyses (e.g., Ghai et al., 2018: stride length g = 0.48) are reported without inflating their scope beyond the studied populations. The framework explicitly does not claim that cueing results in Parkinson’s disease generalize directly to aging; rather, it proposes that the underlying principle may generalize, which is a weaker and more defensible claim appropriate to a Perspective article.

It should be noted that the framework’s falsifiability is contingent on operationalization. If future measurement development reveals that ‘internal reference integrity’ cannot be reliably quantified, or that external reference inputs produce no detectable effects on coordination variables in aging populations, the framework would require significant revision. This vulnerability is a scientific virtue, not a defect: it preserves the framework’s status as a hypothesis rather than an unfalsifiable proposition.

## 9 Research directions and future implications

### 9.1 Operationalizing loss of internal reference

Developing reliable measures of internal reference integrity is essential, but the critical empirical question for this framework is one level higher. Geometric pacing is not defined by its effect on individual-system variability; it is defined by its effect on coordination between systems. The framework’s principal falsifiable prediction is therefore best formulated at the inter-system coupling level, using measures developed within Network Physiology for precisely this purpose (Bashan et al., 2012; Bartsch et al., 2015; Ivanov, 2021; Garcia-Retortillo et al., 2026).

The prediction is as follows. If an appropriate external reference is delivered for a defined exposure window, at least one cross-system coupling link in the cardiorespiratory, respiratory–postural, or cardio–postural subnetwork should show a measurable increase in its strength or temporal stability, quantifiable as a rise in time-delay stability, in wavelet phase coherence, or in amplitude-amplitude cross-frequency coupling (Bashan et al., 2012; Bartsch et al., 2015; Garcia-Retortillo et al., 2026). This change should (a) exceed what is observed under a matched informationally richer but temporally unstructured input; (b) precede any change in subjective symptoms; and (c) be at least partially dissociable from change in the within-system variability of the participating signals. A pattern in which within-system variability shifts without a corresponding change in coupling would be consistent with a within-system entrainment account and would not support the organism-level framing of geometric pacing. The framework therefore stands or falls on a demonstration that the coupling level moves when the system-level signals do.

This prediction is empirically tractable with existing instrumentation. Synchronized multi-channel recording of ECG, respiration, and either accelerometry or force-plate posturography, in windows of approximately 5 minutes and a short-duration crossover design, is sufficient to compute the proposed coupling indices and to test the temporal dissociation at the core of the prediction. Candidate within-system measures—including HRV complexity, gait variability (Hausdorff, 2009), and postural sway—remain useful as secondary endpoints and for characterizing the single-system state of the participating signals.

### 9.2 Identifying effective external references

What forms of reference—rhythmic, spatial, proprioceptive, stochastic, or multimodal—benefit which systems? Are there individual differences in reference responsiveness? What parameters optimize stabilization? The cueing literature provides starting points, but systematic parametric studies are needed. The inclusion of stochastic resonance paradigms alongside periodic cueing studies would be particularly valuable for establishing the comparative efficacy of periodic and non-periodic reference inputs.

### 9.3 Examining interaction effects

Breathing, posture, and autonomic regulation are interrelated. External references targeting one domain may have cascade effects on others. Understanding these interactions is crucial for optimizing future interventions and avoiding unintended consequences.

### 9.4 Assessing recalibration effects

Does prolonged external reference exposure produce lasting changes in internal coordination? If so, what exposure parameters are required, and can benefits transfer to untrained contexts? These questions have practical implications for intervention design and are testable through longitudinal protocols with systematic follow-up assessment.

### 9.5 Translational horizon: supporting pharmacological resilience

A further implication—not as a direct claim of the present manuscript, but as a broader translational horizon for the wider manuscript series—concerns pharmacological resilience (Carballo, 2026). If external reference support can help preserve organism-level coordination before overt pathology consolidates, such approaches may eventually contribute to therapeutic strategies that support function earlier and more coherently, and in selected contexts may reduce escalating dependence on symptom-driven pharmacological compensation.

This possibility should be stated cautiously and tested empirically. Even so, it is consonant with the Network Physiology perspective that interventions should be evaluated not only by their effects on isolated endpoints, but also by how they modify interactions among systems and subsystems (Ivanov, 2021). The full research agenda is outlined in Table 4.

**TABLE 4:** Proposed research agenda for geometric pacing.

Research priority	Key questions	Methodological approaches
Cross-system coupling measurement	Can external references measurably stabilize inter-system coupling, dissociably from within-system variability?	Time-delay stability, wavelet phase coherence, amplitude-amplitude cross-frequency coupling; synchronized multi-channel recordings
Within-system measurement development	How should internal reference integrity be operationalized as a secondary endpoint?	HRV complexity, gait variability, postural sway, multimodal time-series analysis
Parameter optimization	Which reference types and parameters optimize stabilization? (including stochastic vs. periodic)	Parametric studies, adaptive designs, individual-difference analyses
Mechanism investigation	How do external references modify coordination dynamics?	Neuroimaging, computational modeling, perturbation studies
Durability assessment	Does exposure produce lasting recalibration?	Longitudinal designs, follow-up protocols, transfer tests

The first row reflects the R3 reformulation of the framework’s principal falsifiable prediction at the inter-system coupling level.

## 10 Conclusion

Cardiac pacemakers demonstrate that restoring function does not always require correction of cause; sometimes the provision of a stable reference is sufficient to re-enable self-organization. Extending this insight beyond the heart suggests that geometric pacing may represent a broader principle of physiological stabilization.

The present Perspective argues that this principle is best understood within the framework of Network Physiology. Geometric pacing is not aimed at isolated organs but at interactions across systems and subsystems; not at static defects but at dynamic instability; and not at a single time scale but at multiscale physiological integration. It therefore offers a conceptual vocabulary for thinking about aging, persistent physical symptoms, and pre-pathological states as conditions of disturbed organism-level coordination.

Whether this principle ultimately informs rehabilitation, environmental design, wearable systems, or future clinical interventions remains open. What is offered here is not a finished solution but a field-appropriate hypothesis: that external reference support—whether periodic, spatial, proprioceptive, or stochastic in nature—may help stabilize physiological networks when intrinsic coordination becomes fragile.

## Data Availability

The original contributions presented in the study are included in the article/supplementary material, further inquiries can be directed to the corresponding author.
